# Modelling Short-Term Maximum Individual Exposure from Airborne Hazardous Releases in Urban Environments. Part ΙI: Validation of a Deterministic Model with Wind Tunnel Experimental Data

**DOI:** 10.3390/toxics3030259

**Published:** 2015-06-26

**Authors:** George C. Efthimiou, John G. Bartzis, Eva Berbekar, Denise Hertwig, Frank Harms, Bernd Leitl

**Affiliations:** 1 Environmental Research Laboratory, INRASTES, NCSR Demokritos, Patriarchou Grigoriou & Neapoleos Str., 15310 Aghia Paraskevi, Greece; 2 Department of Mechanical Engineering, University of Western Macedonia, Sialvera & Bakola Str., 50100 Kozani, Greece; E-Mail: bartzis@uowm.gr; 3 Meteorological Institute, KlimaCampus, University of Hamburg, Bundesstrasse 55, D-20146 Hamburg, Germany; E-Mails: eva.berbekar@zmaw.de (E.B.); denise.hertwig@zmaw.de (D.H.); frank.harms@zmaw.de (F.H.); bernd.leitl@zmaw.de (B.L.)

**Keywords:** dosage, individual exposure, turbulence integral time scale, wind tunnel measurements, validation

## Abstract

The capability to predict short-term maximum individual exposure is very important for several applications including, for example, deliberate/accidental release of hazardous substances, odour fluctuations or material flammability level exceedance. Recently, authors have proposed a simple approach relating maximum individual exposure to parameters such as the fluctuation intensity and the concentration integral time scale. In the first part of this study (Part I), the methodology was validated against field measurements, which are governed by the natural variability of atmospheric boundary conditions. In Part II of this study, an in-depth validation of the approach is performed using reference data recorded under truly stationary and well documented flow conditions. For this reason, a boundary-layer wind-tunnel experiment was used. The experimental dataset includes 196 time-resolved concentration measurements which detect the dispersion from a continuous point source within an urban model of semi-idealized complexity. The data analysis allowed the improvement of an important model parameter. The model performed very well in predicting the maximum individual exposure, presenting a factor of two of observations equal to 95%. For large time intervals, an exponential correction term has been introduced in the model based on the experimental observations. The new model is capable of predicting all time intervals giving an overall factor of two of observations equal to 100%.

## 1. Introduction

The capability to predict short-time maximum individual exposure is very important in order to deal with the release of airborne hazardous materials. Such a quantity is of stochastic nature and practically unpredictable, especially for very small time intervals since the instantaneous conditions of the atmosphere are unknown at the time of the release. However, a parameter very important to emergency management and predictable at the same time is the maximum expected individual exposure, which is defined as the dosage over a specified time interval Δτ:

(1) $$D_{\max}(\Delta \tau )={\left[\int_{0}^{\Delta \tau }C(t)\;dt\right]}_{\max}=C_{\max}\left(\Delta \tau \right)\text{ Δτ}$$

where *C(t)* is the instantaneous concentration at a receptor point, and *C_max_(*Δτ*)* is the maximum time-averaged (peak) concentration over Δτ.

The common methodology today to predict maximum concentrations is the utilization of well-established probability density functions (pdf) (e.g., chopped normal, log-normal, gamma or Weibull) for the concentration distributions [1,2,3,4,5]. In this case, a computational dispersion model uses the predicted concentration mean, variance and intermittency factor and a predefined probability density function as mentioned above, to estimate the peak concentration with a confidence interval (e.g., 95% or 99%). For example, the widely used puff model SCIPUFF [6] uses the chopped normal distribution.

It is to be noted that the results are expected to be sensitive to the particular pdf and especially on the confidence limit selected. Therefore additional criteria are needed to specify the appropriate confidence interval level [7]. It should also be noted that if, theoretically, the confidence limit tends to unity, the peak concentration would tend to infinity. In reality, however, the peak concentration is finite.

Despite the above mentioned difficulties, the challenge remains to build an individual exposure prediction capability in air dispersion models (e.g., Computational Fluid Dynamics models) as simple as possible, dealing with geometries as complex as possible. Along this direction, Bartzis *et al.* (2008) [8] have proposed a relatively simple model based on the hypothesis that key parameters in defining the maximum dosage are the concentration fluctuation intensity (*I*) and the autocorrelation time scale (*Τ_C_*)*,* as follows:

(2) $$D_{\max}(\text{Δτ})=\bar{C}\;\left[1+\text{β }Ι\;{\left(\frac{\text{Δτ}}{T_{\text{C}}}\right)}^{-n}\right]\text{ Δτ}$$

(3) $$I=\frac{\bar{{C^{\prime }}^{2}}}{{\bar{\text{C}}}^{2}}$$

where $\bar{{C^{\prime }}^{2}}$ is the concentration variance and $\bar{C}$ is the mean concentration. These quantities as well as the concentration autocorrelation time scale can be estimated from experimental time series as described in Part I. The extensive analysis of the MUST field concentration data of various stability classes has suggested an indicative value of *β* = 1.72 while the parameter *n* remained the same as the indicative value (=0.3).

The estimation of the parameter *β* based on field data analyses includes uncertainties due to the fact that the concentration time series are subject to the non-stationarity of the ambient atmospheric conditions. Such drawbacks can be eliminated by reverting to reference data, measured in boundary-layer wind tunnels under stationary and well-defined boundary conditions. In the following section, the methodology will be validated against an extensive laboratory dataset obtained from time-resolved concentration measurements in a semi-complex urban model. Using the large amount of available wind-tunnel data provides ways for a more reliable estimation of model uncertainties.

## 2. The Wind Tunnel Measurements

### 2.1. Description of the Concentration Measurements

**Figure 1:**
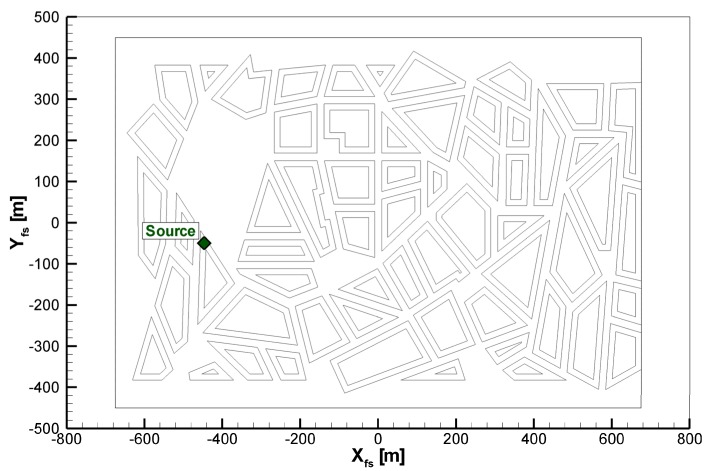
Layout of the Michelstadt model indicating the source location. Flow is approaching from the left.

The measurements considered for model validation were carried out in the “WOTAN” boundary-layer wind tunnel of the Environmental Wind Tunnel Laboratory at the University of Hamburg. The so-called “Michelstadt”, an idealized model of a Central-European city district, has provided the geometric test case in which dispersion measurements were carried out. The model consists of building rings with flat roofs of different heights (15 m, 18 m and 25 m in full scale) forming street canyons with widths of 18 m and 24 m. The properties of the approach flow correspond to a very rough urban boundary layer of neutral stability. Tracer gas was emitted continuously from a point source placed on a rooftop (Figure 1). The concentration was measured using a fast Flame Ionization Detector (FID) at 196 locations between and above the building models of Michelstadt. The measurement was carried out for 275 s at each location to ensure the statistical representativeness of the data. Given the scale-reduced nature of the model (built on a scale of 1:225), this measurement time corresponds signal duration of more than 17 h under full-scale conditions.

### 2.2. Evaluation of the Concentration Data

As previously mentioned, the selected test case refers to a 1:225-scale wind-tunnel model of a semi-idealized urban complexity (Michel-Stadt) that is part of the online validation data base CEDVAL-LES (http://www.mi.zmaw.de/index.php?id=433). All available data of the “Michelstadt” wind-tunnel experiments are included in the validation. The dataset contains high-resolution concentration time series with a sampling time interval of Δτ = 0.005s from 196 fast FID sensor measurements. Each sensor contains 55,000 concentration measurements (*i.e.*, 1.078 × 10^7^ data points for all sensors) and at each sensor measurements were taken over a duration of *T* = 275 s. Since the quality and statistical representativeness of the data was regularly verified by repetitive measurements, no outlier-exclusion has been carried out before the model validation. The ratio of minimum and maximum values of the fluctuation intensity (*I_max_*/*I_mean_*) and the ratio of maximum and mean values of concentration (*C_max_*/*C_mean_*) are 0.077/15.57 and 16.55/8.82 respectively. Following Yee and Biltoft, (2004) [9] the autocorrelation time *T_C_* is calculated from the autocorrelation function *R_C_(*τ*)* until the value at *R_C_(*τ*)* first decreases below 0.1. The min/max values of *T_C_* were determined to be 0.041 s/1.001 s, which correspond to a normalized time range *T/T_C_* = 275 to 6707. These values are long enough to assume turbulence stationarity.

## 3. Model Refinement and Uncertainties

### Estimation of the Uncertainty of the β Parameter

**Figure 2:**
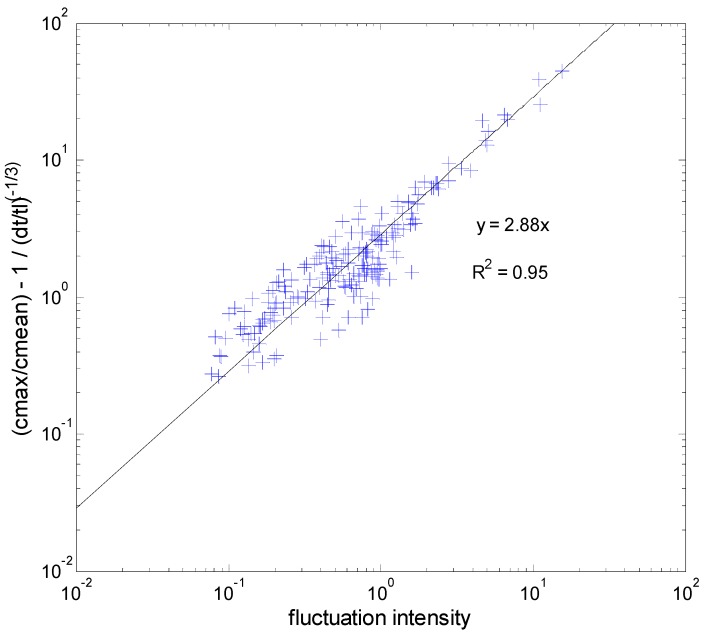
Correlation between the quantity Equation (4) and the fluctuation intensity (*I*). The data follow a linear relationship with a slope of 2.88 and a correlation coefficient *R^2^* = 0.95.

**Figure 3:**
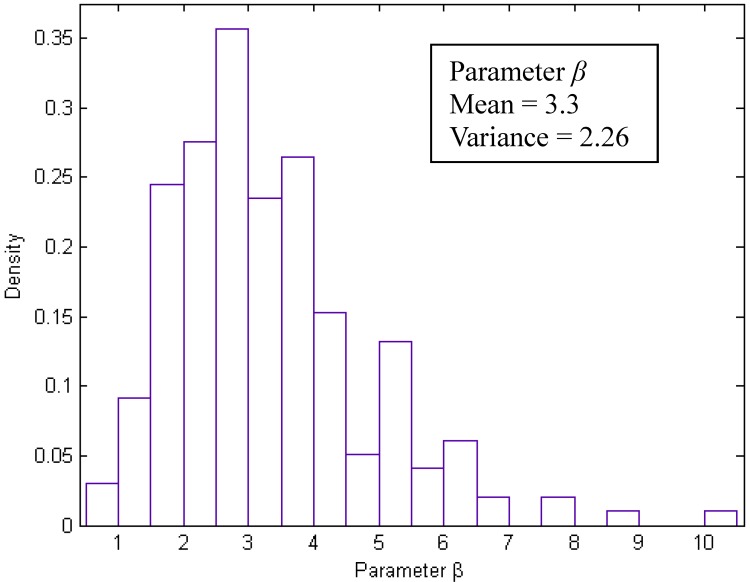
The probability density function of the parameter *β*.

Following the same procedures as in Part I, for the model refinement, only high resolution (Δτ*_0_* = 0.005s) data has been considered. For the estimation of the parameter *β*, the exponent *n* was kept constant (*n* = 0.3) whereas the parameter *β* was varied from signal to signal. The decision of fixing the *n* value is based on the experimental evidence of relatively mild variability [8]. On the other hand, any variability of *n* will be absorbed on further variability of the *β* parameter. Thus in this case the imperfectness of the model, as well as possible statistical reproducibility of the measurement results, is going to be reflected in the variability of the *β* value. The indicative value of the constant *β* is obtained from a best fit analysis of Equation (4) versus *I* where:

(4) $$\left[\frac{{\text{C}}_{\text{max}}\left(\Delta \tau \right)}{\bar{\text{C}}}-1\right]/{\left(\frac{\text{Δτ}}{{\text{T}}_{\text{C}}}\right)}^{-n}$$

as shown in Figure 2. This analysis produces an indicative value of *β* equal to 2.88. This value is higher than the value of 1.72 derived from field data. Τhe upper bound of *β* for the wind-tunnel data was found to be equal to 10 corresponding to a maximum value of *β_max_* ≈ 3.5 × *β*. It should be noticed that only a single value out of 196 (i.e., 0.5%) is above 9. In Figure 3 the probability density function of the parameter *β* is presented with its mean value and variance. It is clear from the histogram that the indicative value of 2.88 lies in the neighborhood of the most probable *β*-value. On the other hand, the vast majority of *β*-data lie below 6.6, *i.e.*, 2.3 × β. The latter value is more representative of the *β* uncertainty in this case.

## 4. Performance of the Model for Δτ = Δτ*_0_*

In Table 1 the factor of two of observations (FAC2) is presented using the two values for the *β* parameter as estimated from experiments: 1.72 (field data) and 2.88 (wind-tunnel data).

**Table 1:** *C_max_* model versus observation performance for Δτ = Δτ*_0_*.

Parameter *β*		FAC2
Original model	1.72	81.63%
Present model	2.88	95.41%

## 5. The Model’s Overall Performance (Δτ ≥ Δτ*_0_*)

Next to the estimation of the model parameters (Εquation (2), there is also a need to test the performance of the model for large time intervals. Thus, the model performance is tested with the wind-tunnel data for time intervals ranging from Δτ*_0_* = 0.005s to 10s (Δτ*/*Δτ*_0_* = 1–2000).

In Table 2 the factor of two of observations (FAC2) is presented for Δτ ranging from Δτ*_0_* to 10 s using the two values for the parameter *β*.

**Figure 4:**
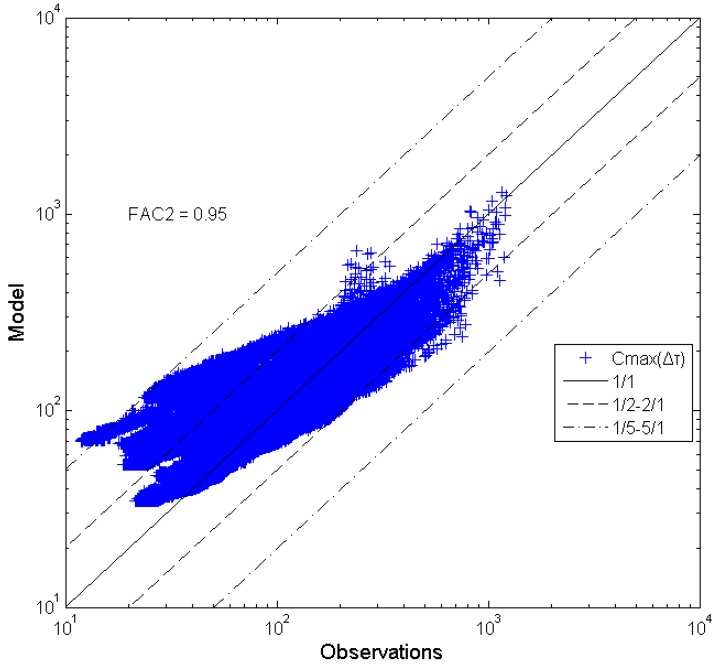
Peak concentration comparisons (Δτ*/*Δτ*_0_* = 1–2000).

In this case the original value of 1.72 gives better results for large Δτ values. It is surprising that FAC2 is slightly smaller for *β* = 2.88. In Figure 4, all *C_max_(*Δτ*)* data are compared with the model equation (2) with *β* = 2.88. It can be observed that the model predicts the experimental values at small integral times rather well, while a model overprediction occurs for the large time intervals (the beta dependence on Δτ*/T_C_* is examined in the next section). It is obvious that there is a need for model improvements with the following two characteristics:

1. For small time integrals the results of the model should be the same as before.
2. For large time integrals the results of the model should be decreased.

**Table 2:** *C_max_* modelversus observation performance for Δτ/Δτ*_0_* = 1–2000.

Parameter *β*		FAC2
Original model	1.72	97.4%
Present model	2.88	95%

## 6. Μodel Improvements

In order to fulfill the above mentioned two characteristics one plausible approach is to allow the *β* parameter to be a function of Δτ*/T_C_* instead of being held constant, *i.e.*,:

(5) $$\text{β}=\text{β}\left(\frac{\text{Δτ}}{{\text{T}}_{\text{C}}}\right)$$

According to the first characteristic (“For small time integrals the results of the model should be the same as before”) the model behavior at small Δτ suggests:

(6) $${\text{β}}_{0}=2.88$$

In order to fulfill the second characteristic (“For large time integrals the results of the model should be decreased”) an extensive data analysis was performed with a view of identifying a proper *β*—function. For a first time the following correlation for the parameter *β* is suggested:

(7) $$\text{β}\left(\frac{\text{Δτ}}{{\text{T}}_{\text{C}}}\right)={\text{β}}_{0}{\text{e}}^{\text{-α}\frac{\text{Δτ}}{{\text{T}}_{C}}}$$

The best-fit analysis of *β* versus Δτ*/T_C_* indicates the following value for *a*:

(8) α=0.012

**Figure 5:**
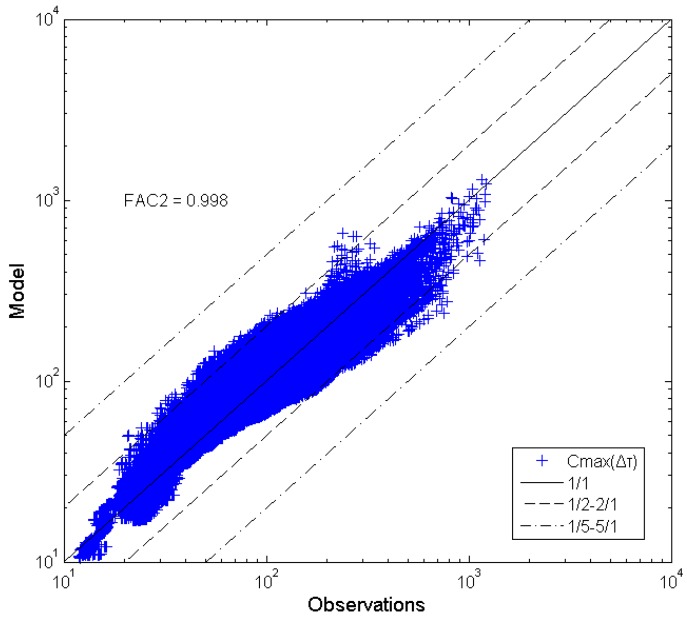
Peak concentration comparisons using the improved model (9) (Δτ*/*Δτ*_0_* =1–2000).

Thus, the improved model for the *C_max_(Δτ)* estimation is given by:

(9) $$C_{\max}(\text{Δτ})=\bar{\text{C}}\left[1+{\text{βe}}^{\text{-a}\frac{\text{Δτ}}{{\text{T}}_{\text{c}}}}I{\left(\frac{\text{Δτ}}{{\text{T}}_{\text{c}}}\right)}^{-n}\right]$$

with *β* = 2.88, *n* = 0.3 and *a* = 0.012. The improved *C_max_(*Δτ*)* model results are presented in Figure 5.

In comparing Figure 4 and Figure 5 it is obvious that the corrected model performs considerably better than the original one. An indicator of this is the increase of the FAC2 to a value of 0.998. Equation (9) will be examined in the future with more wind tunnel data.

## 7. Conclusions

The present work concerns the validation of the Bartzis *et al.* (2008) [8] empirical model for *C_max_(*Δτ*)* to reliably predict the individual maximum exposure in the case of deliberate or accidental releases of hazardous substances into the near-surface atmosphere. For the first time, concentration data from a boundary-layer wind-tunnel experiment in a semi-idealized urban geometry were used as a reference database. The extensive dataset of the “Michelstadt” laboratory experiment carried out under neutral atmospheric stability conditions was analyzed. The dataset contained concentration data from a total of 196 sensors with a sampling time interval of 0.005 s. From the data analysis, the parameter *β* was estimated to be equal to 2.88. With the estimated value of the parameter *β*, the *C_max_(*Δτ*)* model performed very well (FAC2 ≈ 0.95) in predicting the maximum individual exposure. For large time intervals, an exponential correction term has been introduced in order to determine the *β*-value based on experimental observations. For the present dataset, the new model is capable of predicting all time intervals giving an overall FAC2 ≈ 1.
